# Curcumin Inhibits Neuronal and Vascular Degeneration in Retina after Ischemia and Reperfusion Injury

**DOI:** 10.1371/journal.pone.0023194

**Published:** 2011-08-09

**Authors:** Leilei Wang, Chuanzhou Li, Hao Guo, Timothy S. Kern, Kun Huang, Ling Zheng

**Affiliations:** 1 College of Life Sciences, Wuhan University, Wuhan, China; 2 Tongji School of Pharmacy, Huazhong University of Science and Technology, Wuhan, Hubei, China; 3 Department of Medicine, Case Western Reserve University, Cleveland, Ohio, United States of America; 4 Louis Stokes Cleveland Department of Veterans Affairs Medical Center, Cleveland, Ohio, United States of America; University of Oldenburg, Germany

## Abstract

**Background:**

Neuron loss, glial activation and vascular degeneration are common sequelae of ischemia-reperfusion (I/R) injury in ocular diseases. The present study was conducted to explore the ability of curcumin to inhibit retinal I/R injury, and to investigate underlying mechanisms of the drug effects.

**Methodology/Principal Findings:**

Different dosages of curcumin were administered. I/R injury was induced by elevating the intraocular pressure for 60 min followed by reperfusion. Cell bodies, brn3a stained cells and TUNEL positive apoptotic cells in the ganglion cell layer (GCL) were quantitated, and the number of degenerate capillaries was assessed. The activation of glial cells was measured by the expression level of GFAP. Signaling pathways including IKK-IκBα, JAK-STAT1/3, ERK/MAPK and the expression levels of β-tubulin III and MCP-1 were measured by western blot analysis. Pre-treatment using 0.01%–0.25% curcumin in diets significantly inhibited I/R-induced cell loss in GCL. 0.05% curcumin pre-treatment inhibited I/R-induced degeneration of retinal capillaries, TUNEL-positive apoptotic cell death in the GCL, brn3a stained cell loss, the I/R-induced up-regulation of MCP-1, IKKα, p-IκBα and p-STAT3 (Tyr), and down-regulation of β-tubulin III. This dose showed no effect on injury-induced GFAP overexpression. Moreover, 0.05% curcumin administered 2 days after the injury also showed a vaso-protective effect.

**Conclusions/Significance:**

Curcumin protects retinal neurons and microvessels against I/R injury. The beneficial effects of curcumin on neurovascular degeneration may occur through its inhibitory effects on injury-induced activation of NF-κB and STAT3, and on over-expression of MCP-1. Curcumin may therefore serve as a promising candidate for retinal ischemic diseases.

## Introduction

Ischemia contributes to multiple ocular diseases, including glaucoma and diabetic retinopathy [1]. Acute retinal ischemia caused by high ocular pressure followed by reperfusion (I/R injury) leads to neuronal and vascular degeneration, and to inflammatory changes, including up-regulation of TNF-α, COX-2 and iNOS [2], [3]. All of these abnormalities have also been found to be elevated in rodent models of diabetic retinopathy [4], [5], [6], [7], but the changes in retinal I/R injury develop more rapidly and severely. Investigators have used the rodent retinal I/R model to study the mechanisms involved in the neurovascular degeneration and to seek therapeutic ways to prevent this degeneration [2], [3].

The mechanisms triggering retinal neurovascular degeneration are not fully understood. Several pathways have been demonstrated to play important roles in neuronal degeneration after I/R injury, including glutamate excitotoxicity, oxidative and nitrative stress, and inflammation [1]. Previous laboratory and proteomic studies have demonstrated that aminoguanidine, an iNOS inhibitor, inhibits retinal neurovascular degeneration following I/R injury, seemingly via normalization of the expression levels of glycolytic related enzymes [2], [8], [9]. Using CD40 knockout mice, we demonstrated that the deficiency of CD40 gene inhibited I/R-induced neurovascular degeneration, suggesting that the inflammatory and immune systems play critical roles in the development of this I/R-induced retinal injury [3]. However, further studies on the development of retinal neurovascular degeneration are needed not only for better understanding of the pathogenesis of this disease, but also for the discovery of novel therapeutic or preventative methods.

Curcumin is a natural product extracted from turmeric (*Curcuma longa*) that has been used for centuries in Asia to treat various illnesses. Curcumin has been demonstrated to have beneficial effects on rodent models of cancer, diabetes, cardiovascular diseases, arthritis and Alzheimer's disease [10]. The suggested mechanisms underlying these protective effects are based on inhibitory actions of curcumin on disease-mediated induction of inflammatory transcription factors, protein kinases, adhesion molecules, oxidative stress and inflammation (see review of [11]).

The effects of curcumin on retinal diseases have been explored recently. In streptozotocin-induced diabetic rats, curcumin administration has been reported to inhibit a hyperglycemia-induced reduction in antioxidant capacity, and to inhibit diabetes-induced increases in retinal levels of IL-1β, VEGF and NF-κB [12], [13]. In a light-induced retinal degeneration model, curcumin showed neuroprotective effects by inhibiting activation of NF-κB and expression of inflammatory factors [14]. Effects of curcumin on retinal I/R injury, especially on neurovascular degeneration, have not been investigated. To address this, the effects of curcumin on neuronal degeneration, vascular degeneration, glial cell activation, and several signaling pathways/factors involved in inflammatory responses have been evaluated after retinal I/R injury. Our results suggest that curcumin has neuronal and vascular protection effects, but shows no effect on glial activation after retinal I/R injury.

## Materials and Methods

### Ethics Statement

Animal protocols were carried out with approval from the Review Board for animal studies at the Wuhan University (Approval Number #0200909) and according to the guidelines of the Association for Research in Vision and Ophthalmology Statement for the Use of Animals in Ophthalmic and Vision Research, which are also approved by the Committee on Ethics in the Care and Use of Laboratory Animals of Wuhan University.

### Rat model of retinal ischemia-reperfusion

Male Wistar rats (200–300 g) were obtained from the Hubei Animal Laboratory, and housed in ventilated microisolator cages with free access to water and food. Retinal I/R injury was induced as previously described [2], [3]. Briefly, the anterior chamber of one eye was cannulated with a 27-gauge needle attached to an infusion line of normal saline. Pressure in the eye was increased to 80–90 mmHg with a pressure infuser (Infu-surg, Ethox Corp., Buffalo, NY). The other eye of the same animal served as the control. The duration of ischemia was 60 min. After ischemia, the needle was withdrawn, and reflow of the retinal circulation was documented visually. Curcumin powder (Sinopharm Chemical Reagent Co. Ltd., Shanghai, China) was first mixed with the powder form of normal rodent chow in a food mixer for 2–3 hours, and then the curcumin-containing chow was pelleted. Food pellets without curcumin were used as the control diet. Curcumin (0.01%, 0.05% and 0.25%, which are equivalent to 100, 500 and 2500 ppm in diets) was administered to rats 2 days before the injury (for the prevention studies). For another group of rats, 0.05% curcumin was administered 2 days after the injury (for the intervention studies). Animals were sacrificed at 12 hours to 7 days after I/R injury for different analyses.

### Isolation of retinal vasculature and quantitation of degenerate capillaries

Eyes were isolated as previously described [2], [4], [6] and fixed with 10% neutral buffered formalin. Retinas were isolated, washed in water overnight, and then incubated with 3% Difco crude trypsin (BD BioSciences, Sparks, MD) at 37°C for 2 h. Non-vascular cells were gently brushed away from the vasculature. The isolated vasculature preparations were laid out on slides, and stained with periodic acid-schiff and hematoxylin (PASH). Pictures were taken on the middle and peripheral parts of retinal vascular tree for at least 25 different fields per sample under 200X magnification. Acellular (degenerate) capillaries identified as capillary-sized vessel tubes (>30% diameter of regular capillary) that have no nuclei anywhere along their length, were quantitated using the ImagePlus 6.0 software (Media Cybernetics, Bethesda, Maryland) and reported per square millimeter of retinal area. 6–10 rats per group were used for this analysis.

### Examination of cell loss in ganglion cell layer

Enucleated eyes were routinely fixed in 10% buffered formalin, embedded in paraffin, sectioned, and stained with PASH. High resolution (×400 magnification) pictures of 6 different areas per retina with about 340 µm retinal length per area (three continuous pictures from each side of optic nerve head) were taken under an Olympus BX60 microscope equipped with a digital CCD. The nuclei in the ganglion cell layer (GCL; not including nuclei in the vessels), which include ganglion cells and displaced amacrine cells [15], were counted using the ImagePlus 6.0 software. 6–8 rats per group were used for this analysis.

### TUNEL assay

TUNEL assay (In Situ Cell Death Detection kit, fluorescein; Roche, Mannheim, Germany) was performed to detect apoptotic cell death on the paraffin-embedded retinal sections, as we described previously [2]. TUNEL positive cells in the GCL, inner nuclear layer (INL) and outer nuclear layer (ONL) were counted at 6 different areas per retinal section as described above, and reported as number of TUNEL positive cells per millimeter of retinal surface. 5–6 samples per group were used for this analysis.

### Immunofluorescence staining

Paraffin-embedded sections were deparaffinized in xylene and rehydrated in decreasing concentrations of ethanol. After blocking with 2% goat serum, anti-β-tubulin III (1∶500 dilution, Millipore, Eschborn, Germany), anti-brn3a (1∶50 dilution, Millipore, Eschborn, Germany) and GFAP (1∶150 dilution, Millipore) antibodies were applied on the sections for 1 h at room temperature. After washing, the slides were then incubated with the respective secondary antibodies for 1 h. After extensive washing with PBS, sections were imaged by fluorescence microscopy. To measure the brn3a stained cell loss, pictures were taken 6 different areas per retinal section as described above, and brn3a stained cells were counted. The result was reported as number of Brn3a stained cells per millimeter of retinal surface. 4–6 samples per group were used for brn3a and GFAP staining.

### Western blot analysis

Retinas were isolated and sonicated in RIPA buffer (Beyotime Biotech, China). Protein concentration was determined with the BCA protein assay (Beyotime Biotech). 20–50 µg protein per sample was separated by SDS-PAGE and electroblotted onto PVDF membrane (Millipore), and membranes were then stained with washable Ponceau S solution to confirm equal protein loading. After washing, membranes were blocked in Tris-buffered saline with 0.02% Tween 20 and 5% nonfat milk. Antibodies for β-tubulin III (1∶5000 dilution, Beyotime Biotech), GFAP (1∶2000 dilution, Beyotime Biotech), IKKα (1∶1000 dilution, CST, Danvers, MA), IKKβ (1∶1000 dilution, CST), IKKγ (1∶400 dilution, Santa Cruz Biotechnology, Santa Cruz, CA), IκBα (1∶1000 dilution, CST), p-IκBα (1∶500 dilution, Santa Cruz Biotechnology), STAT3 (1∶1000 dilution, CST), p-STAT3-Tyr (1∶1000 dilution, CST), p-STAT3-Ser (1∶1000 dilution, CST), STAT1 (1∶1000 dilution, CST), p-STAT1 (1∶1000 dilution, CST), MCP-1 (1∶500 dilution, Santa Cruz Biotechnology), ERK (1∶5000 dilution, CST), p-ERK (1∶1000 dilution, CST) and β-actin (1∶10000 dilution, Sigma, St. Louis, MO) were applied overnight at 4°C. All blots were washed and incubated with respective horseradish peroxidase coupled secondary antibodies (Bio-Rad) at a dilution of 1∶1000–1∶10000. After extensive washing, protein bands detected by the antibodies were visualized by ECL reagent followed by exposure on X-OMAT film. The films were subsequently scanned and band intensities were quantified using the Quantity One 1-D Analysis Software (Bio-Rad). The protein levels, except for the phosphorylated proteins, were first quantitated relative to β-actin in the same sample, and then the relative protein expression levels in different groups were normalized to the non-injured group which set up as 1 fold. Whereas the levels of the phosphorylated proteins were first quantitated relative to their corresponding total protein levels in the same sample, and then the relative phosphorylated protein expression levels in different groups were normalized to the non-injured group which set up as 1 fold. Each western blot analysis was repeated 2–3 times with 3–5 samples in each group.

### Statistical analysis

All results were expressed as the mean ± SEM (stand error of the mean). Data was analyzed by the nonparametric Kruskal-Wallis test followed by the Mann-Whitney test. Differences were considered statistically significant when the P<0.05.

## Results

**Figure 1:**
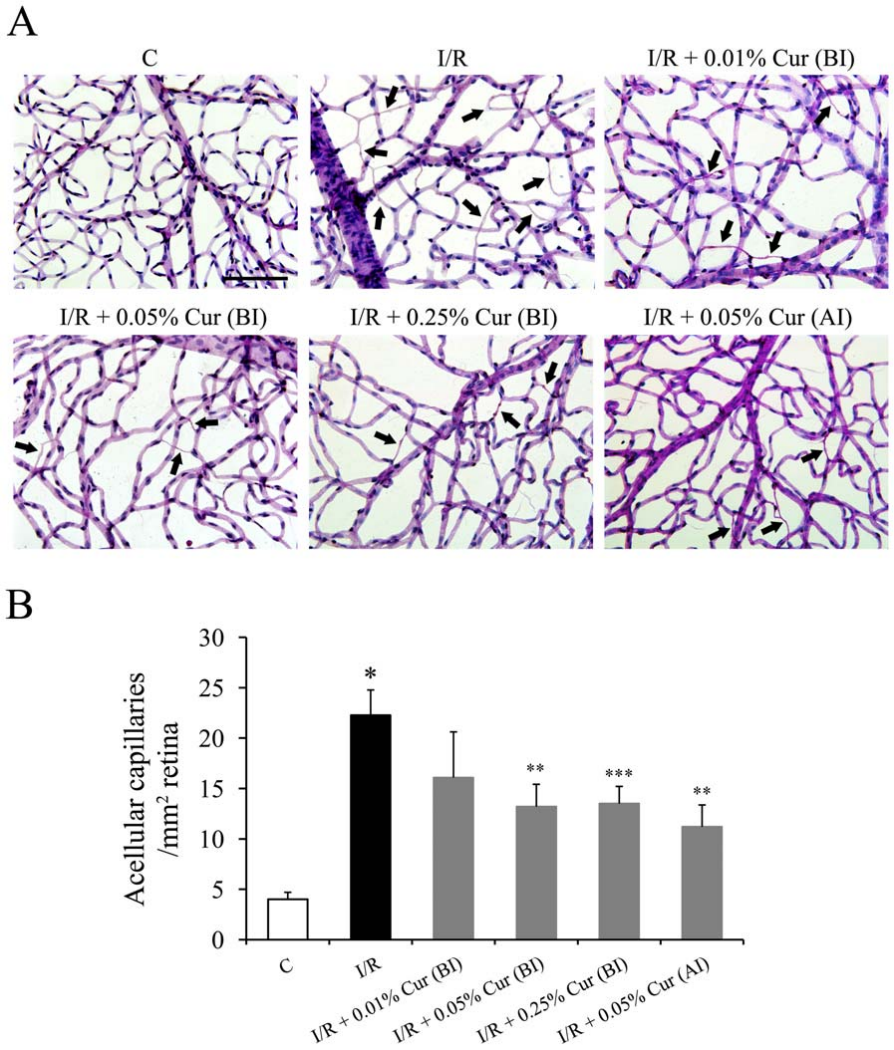
Curcumin partially inhibited retinal I/R induced capillary degeneration. (A) Representative pictures of PASH stained retinal vasculatures isolated from retinas 7 days after the I/R injury. Degenerate capillaries are indicated with black arrowheads. (B) Inhibition of retinal I/R-induced capillary degeneration by curcumin. Group abbreviations are: C, non-injured eyes; I/R, I/R-injured retinas; I/R +0.01% Cur (BI), I/R-injured retinas of animals treated with 0.01% curcumin 2 days before the injury; I/R +0.05% Cur (BI), I/R-injured eyes of animals treated with 0.05% curcumin 2 days before the injury; I/R +0.25% Cur (BI), I/R-injured eyes of animals treated with 0.25% curcumin 2 days before the injury; and I/R +0.05% Cur (AI), I/R-injured eyes of animals treated with 0.05% curcumin 2 days after the injury. (n = 6–10 rats per group; *P<0.001 compared with non-injured retinas; **P<0.01 compared with I/R-injured retinas; ***P<0.05 compared with I/R-injured retinas. The error bars represent standard error of the mean. The scale bar in Fig A represents 100 µm).

As we reported previously, retinal I/R injury induced a 5.6-fold increase in formation of acellular (degenerate) capillaries in the injured retinas compared to non-injured retinas 7 days after the injury. Oral administration of 0.05% and 0.25% curcumin before the I/R injury significantly inhibited I/R-induced vascular degeneration in the retina by approximately 40%, whereas 0.01% curcumin showed no significant protective effect on this lesion (Figure 1). Oral administration of 0.05% curcumin 2 days after I/R injury (when neuronal degeneration already was extensive) still showed a significant vascular protective effect on the vasculature (Figure 1).

**Figure 2:**
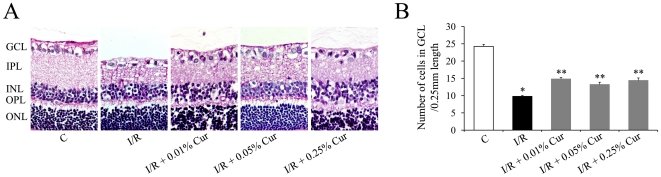
Curcumin partially inhibited retinal I/R induced neuronal degeneration. (A) Representative pictures of PASH stained retinal sections 2 days after the injury (100 µm length per picture). (B) Number of cells in the GCL was measured as described in Methods. Group abbreviations are: C, non-injured eyes; I/R, I/R-injured eyes; I/R +0.01% Cur, I/R-injured eyes of animals treated with 0.01% curcumin 2 days before the injury; I/R +0.05% Cur, I/R-injured eyes of animals treated with 0.05% curcumin 2 days before the injury; I/R +0.25% Cur, I/R-injured eyes of animals treated with 0.25% curcumin 2 days before the injury. (n = 6–8 rats per group; *P<0.001 compared with non-injured retinas; **P<0.005 compared with I/R-injured retinas). The error bars represent standard error of the mean.

Two days after retinal I/R injury, there was a significant cell loss in the GCL, which is consistent with our previous observations [2]. Oral administration of 0.01%, 0.05% and 0.25% curcumin all significantly inhibited I/R-induced cell loss in GCL (Figure 2). Inhibition of I/R-induced cell loss in GCL by curcumin in our study did not show a dose-related effect, but neuronal cells were protected at lower doses of curcumin than were vascular cells. 0.05% curcumin was selected for further studies since it was the lowest dosage that showed both vascular and neuronal protective effects on the injury.

**Figure 3:**
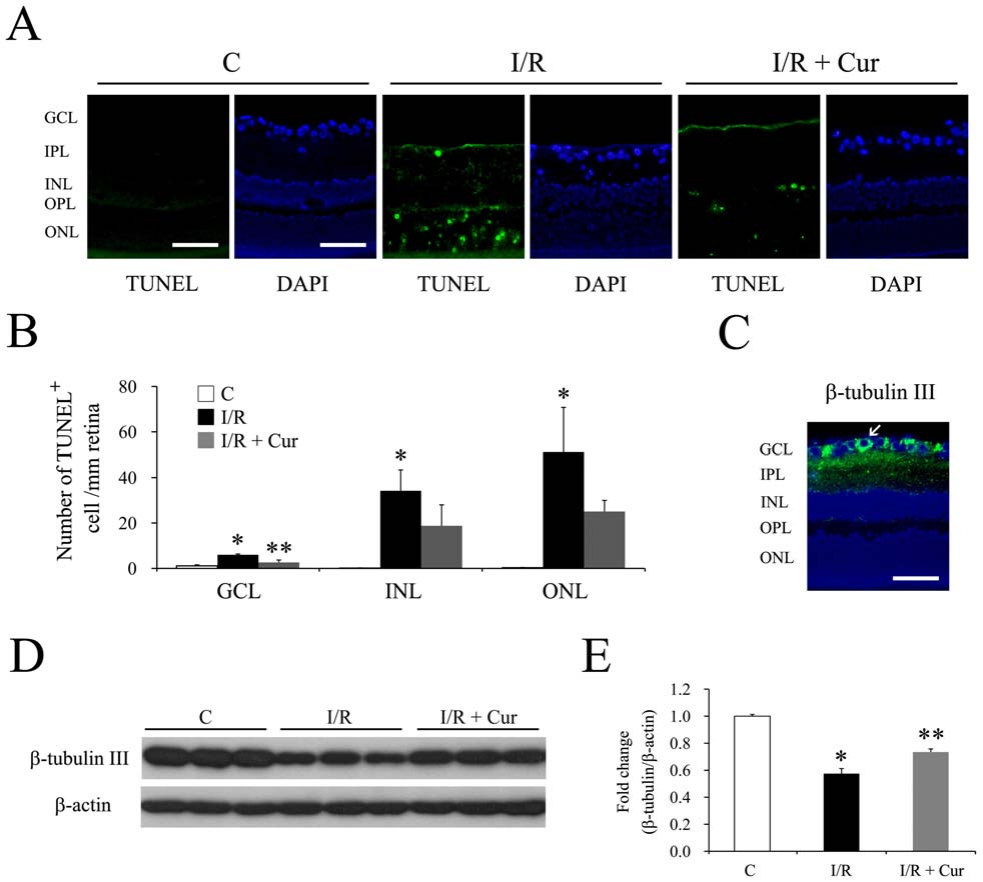
Curcumin inhibited I/R induced apoptotic cell death of retinal neurons and down-regulation of β-tubulin III. (A) Representative pictures of TUNEL assay on the retina sections 2 days after the injury. A TUNEL-positive retinal ganglion cell is indicated with white arrowhead. Blue color: DAPI stained nuclei. Green color: TUNEL-positive cells. The scale bar represents 50 µm. (B) Quantitative results of TUNEL-positive cells in the GCL. (n = 4–5 different samples in each group, *P<0.01 compared with non-injured retinas; **P<0.05 compared with I/R-injured retinas) The error bars represent standard error of the mean. (C-E) Curcumin rescued retinal I/R-induced down-regulation of β-tubulin III 2 days after I/R injury. (C) Immunofluorescence staining of β-tubulin III on retinal sections. Blue color: DAPI stained nuclei. Green color: β-tubulin III staining. The scale bar represents 50 µm. Representative western blots of β-tubulin III and β-actin in retinas (D) along with densitometric quantitative results (E) (n = 4 different samples in each group, *P<0.05 compared with non-injured retinas; **P<0.05 compared with I/R-injured retinas). The error bars represent standard error of the mean. Group abbreviations are: C, non-injured eyes; I/R, I/R-injured eyes; I/R + Cur, I/R-injured eyes of animals treated with 0.05% curcumin 2 days before the injury.

Apoptosis contributes to retinal cell loss after retinal I/R injury. TUNEL assay was performed to measure the apoptotic cell death in retinal sections 2 days after the injury. Our results indicate that I/R injury caused significant increases of TUNEL-positive cells in the GCL, INL and ONL. 0.05% curcumin pre-treatment inhibited retinal I/R induced apoptotic cell death in the GCL, but not in the other two layers (Figure 3A and 3B).

**Figure 4:**
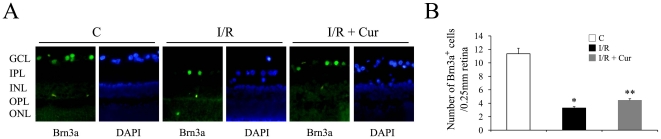
Curcumin partially inhibited retinal I/R-induced brn3a stained cell loss. (A) Representative pictures of brn3a stained retinal sections 2 days after the injury (100 µm length per picture). (B) Number of brn3a stained cells was measured as described in Methods. Group abbreviations are: C, non-injured eyes; I/R, I/R-injured eyes; I/R + Cur, I/R-injured eyes of animals treated with 0.05% curcumin 2 days before the injury. (n = 5–6 rats per group; *P<0.01 compared with non-injured retinas; **P<0.02 compared with I/R-injured retinas). The error bars represent standard error of the mean.

There are ganglion cells and displaced amacrine cell in the GCL, so the increased cell loss and TUNEL positive cells in the GCL we observed may come from both cell types. To demonstrate whether curcumin has effects on the retinal I/R induced ganglion cell loss, two different methods were used. β-tubulin III has been reported as a marker of retinal ganglion cells [16], [17], [18], and the immunofluorescence staining also confirmed its cytoplasmic localization in the GCL (Figure 3C). 0.05% curcumin showed ganglion cell protective effects on retinal I/R injury, which was demonstrated by evaluating the expression level of β-tubulin III 2 days after the injury using western blot. As shown in Figures 3D and 3E, retinal I/R injury caused a 1.7-fold reduction on the expression level of β-tubulin III, while curcumin pre-treatment significantly inhibited this I/R-induced down-regulation. Brn3a is a POU domain transcription factor that specifically expressed in the nuclei of retinal ganglion cells [15], [19]. As demonstrated in Figure 4A, brn3a stained nuclei were exclusively located in the GCL, which is consistent with previous reports [15], [20], but only some of the nuclei in the GCL were brn3a stained. Retinal I/R injury induced a 4-fold reduction of brn3a stained cell in the GCL, while curcumin pre-treatment mildly, but significantly inhibited this I/R-induced ganglion cell loss (Figure 4B).

**Figure 5:**
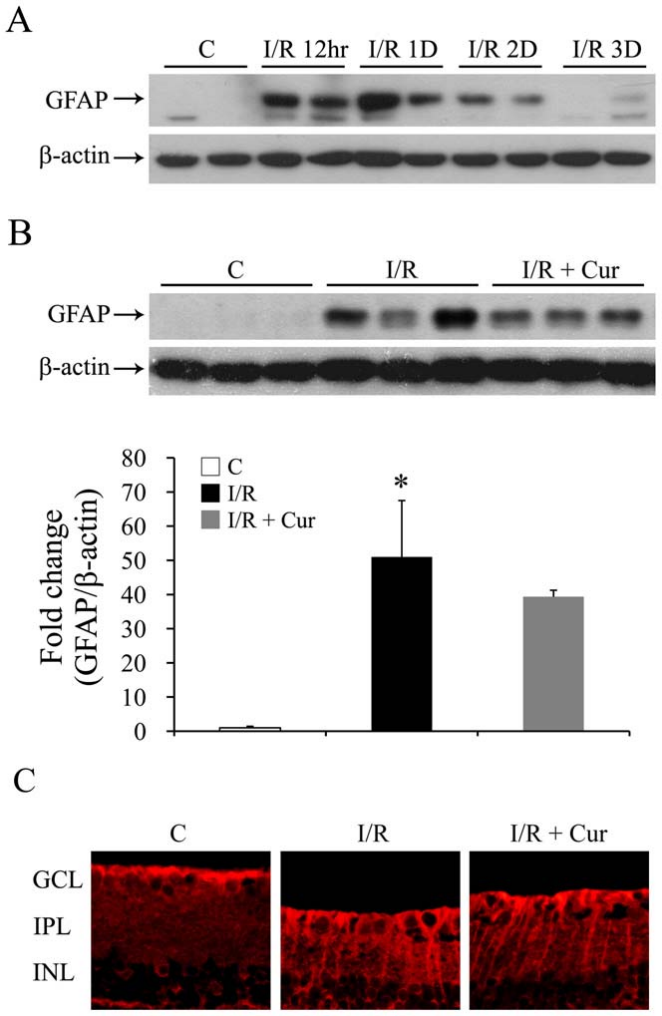
Curcumin showed no effect on I/R induced glial cell activation. (A) Representative Western blots of retinal GFAP expression level 12 hours (12hr), 1 day (1D), 2 days (2D), and 3 days (3D) after retinal I/R injury (top). β-actin in the same samples (bottom) was used as loading control. (B) Representative Western blot of GFAP and β-actin levels in retinas (top) with densitometric quantitative results (bottom) 2 days after retinal I/R injury. (n = 3–5 in each group; *P<0.05 compared with non-injured retinas). The error bars represent standard error of the mean. (C) Representative pictures of GFAP staining on retinal sections 2 days after retinal I/R injury (100 µm length per picture). Group abbreviations are: C, non-injured eyes; I/R, retinal I/R-injured eyes; I/R + Cur, I/R-injured eyes of animals treated with 0.05% curcumin 2 days before the injury.

Glial cell activation is commonly found after retinal injury [21], and the up-regulation of GFAP expression is one of the earliest hallmarks of reactive gliosis [21]. As shown in Figure 5A, increased expression of GFAP was found 12 hours after I/R injury, and this upregulation lasted for at least 3 days. However, administration of 0.05% curcumin did not inhibit the injury-induced GFAP over-expression at either 12, 24 or 48 hrs after the injury (Figure 5B, data for 12 and 24 hrs not shown). Consistent with the western blot results (Figure 5B), administration of 0.05% curcumin showed no effect on the injury-induced GFAP over-expression of retinal sections, which is mainly increased in activated muller cells after the injury (Figure 5C).

**Figure 6:**
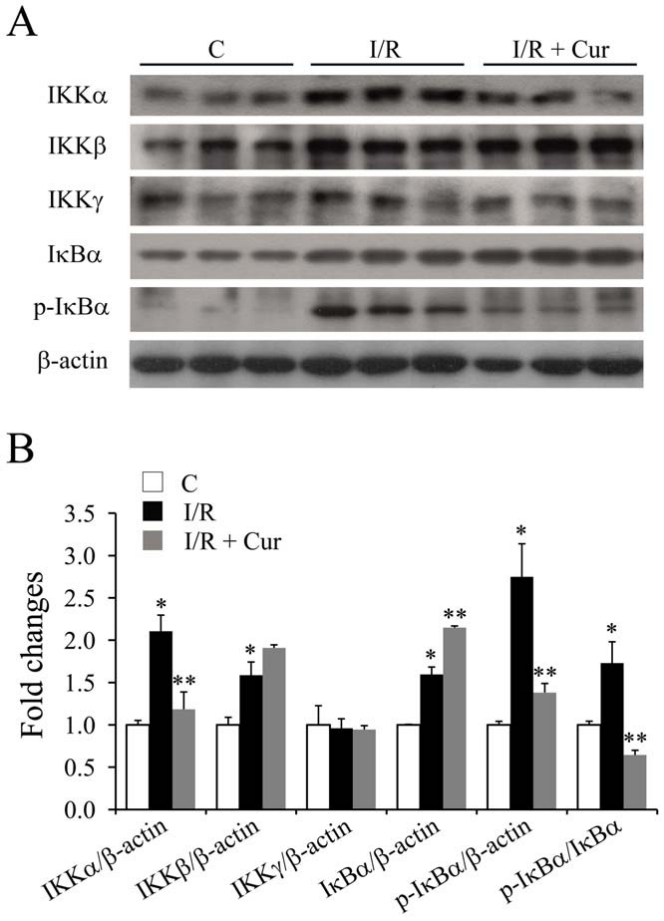
Curcumin inhibited the activation of the NF-κB signaling pathway 2 days after retinal I/R injury. Representative western blots of IKKα, IKKβ, IKKγ, IκBα, p-IκBα and β-actin expression levels in retinas are shown (A) with densitometric quantitative results (B). Group abbreviations are: C, non-injured eyes; I/R, retinal I/R-injured eyes; I/R + Cur, I/R-injured eyes of animals treated with 0.05% curcumin 2 days before the injury. (n = 3–4 in each group; *P<0.05 compared with non-injured retinas; **P<0.05 compared with I/R-injured retinas). The error bars represent standard error of the mean.

Since inflammatory changes can contribute to neurovascular degeneration after retinal I/R injury [2], [8], we proposed that the beneficial effects of curcumin on neurovascular degeneration was due to its ability to inhibit these inflammatory responses. To test this hypothesis, the signaling pathway that regulates activation of NF-κB (a key transcription factor involved in the regulation of inflammation) was investigated 2 days after the injury. Retinal I/R injury induced significant increases in the expression levels of α and β subunits of the IKK (IκB kinase) complex, but not the γ subunit (Figure 6). Our results demonstrated that oral administration of 0.05% curcumin significantly inhibited the up-regulation of IKKα, without affecting the expression level of IKKβ, after I/R injury (Figure 6). The levels of IκBαand p-IκBα were also investigated. Retinal I/R injury caused a mild increase in the total IκBα level, and a dramatic increase in the p-IκBα level. Oral administration of curcumin significantly inhibited the I/R induced phosphorylation of IκBα (Figure 6).

**Figure 7:**
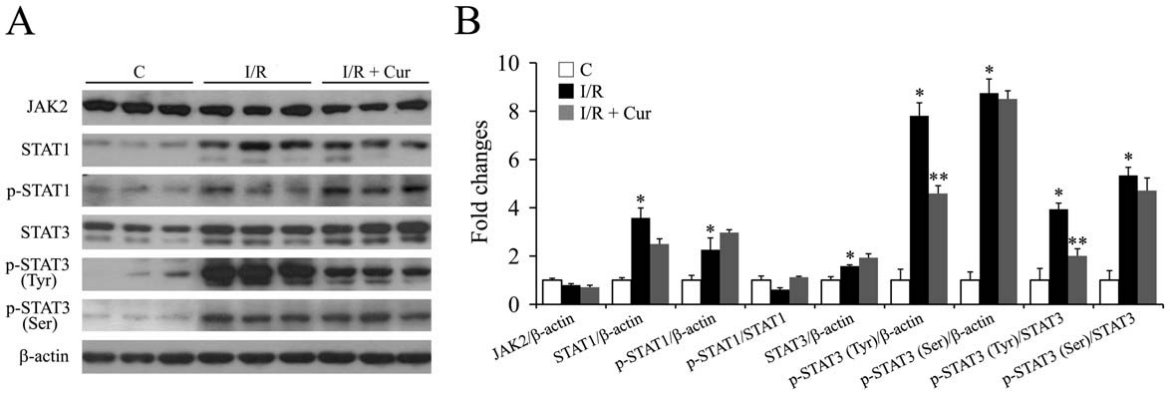
Curcumin inhibited the activation of STAT3, but not STAT1 levels 1 day after retinal I/R injury. Representative Western blots of total JAK2, total STAT1 and p-STAT1, total STAT3, p-STAT3 (Tyr705), p-STAT3 (Ser727) and β-actin show expression levels in retinas (A) with densitometric quantitative results summarized in (B). Group abbreviations are: C, non-injured eyes; I/R, retinal I/R-injured eyes; I/R + Cur, I/R-injured eyes of animals treated with 0.05% curcumin 2 days before the injury. (n = 4–5 in each group; *P<0.02 compared with non-injured retinas; **P<0.01 compared with I/R-injured retinas). The error bars represent standard error of the mean.

STAT (signal transducer and activator of transcription) is another inflammatory regulating transcription factor family, in which STAT1 and 3 are two members. The transcriptional activation of STAT1/3 is regulated by their respective phosphorylation levels. The expression levels of total and phosphorylated STAT3 (p-Tyr705, p-Ser727) and STAT1 (p-Tyr701), as well as JAK2 (the upstream kinase regulating the phosphorylation levels of STAT1/3) were investigated one day after the injury. Our results indicated that retinal I/R injury caused a mild, but significant, increase in total STAT3 (1.6-fold), and dramatic increases in its phosphorylation at sites p-Tyr705 (7.8-fold) and p-Ser727 (8.8-fold) (Figure 7). Oral administration of 0.05% curcumin significantly inhibited the injury-induced over-expression of p-STAT3 (Tyr705), while showed no significant effects on the levels of total STAT3 or p-STAT3 (Ser727). Retinal I/R injury also caused dramatic increases in the levels of total STAT1 (3.6-fold) and p-STAT1 (2.3-fold), but oral curcumin had no significant effects on either (Figure 7). Neither injury nor curcumin pre-treatment showed any effect on the expression level of JAK2 (Figure 7).

**Figure 8:**
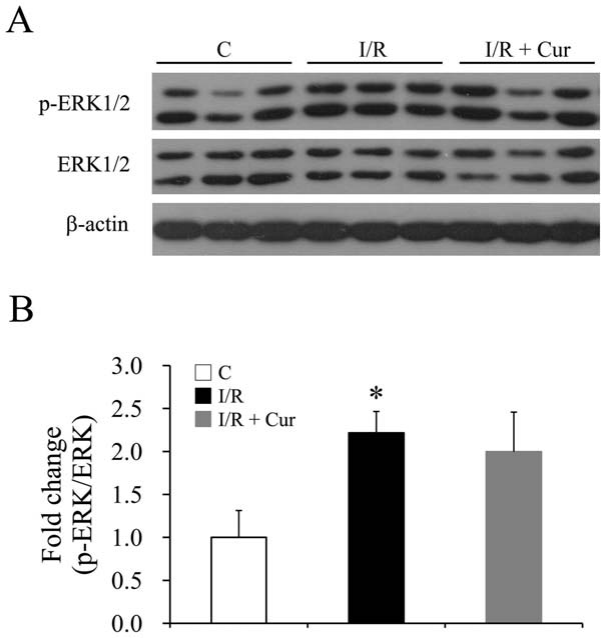
Curcumin showed no inhibitory effect on ERK activation 1 day after retinal I/R injury. Representative western blots of total ERK1/2, phosphorylated ERK1/2 and β-actin (A) with densitometric quantitative results (B). Phosphorylation levels of ERK1/2 were reported relative to total ERK1/2 expression, and normalized to the non-injured group. Group abbreviations are: C, non-injured eyes; I/R, retinal I/R-injured eyes; I/R + Cur, I/R-injured eyes of animals treated with 0.05% curcumin 2 days before the injury. (n = 4–5 in each group; *P<0.05 compared with non-injured retinas). The error bars represent standard error of the mean.

The ERK/MAPK pathway regulates cell survival, proliferation, inflammation and the immune system under multiple conditions. It has been reported that ERK is one of kinases responsible for the Ser727 phosphorylation of STAT3 [22]. Retinal I/R injury significantly up-regulated ERK phosphorylation one day after the injury, but 0.05% curcumin pre-treatment did not inhibit the injury-induced ERK activation (Figure 8).

**Figure 9:**
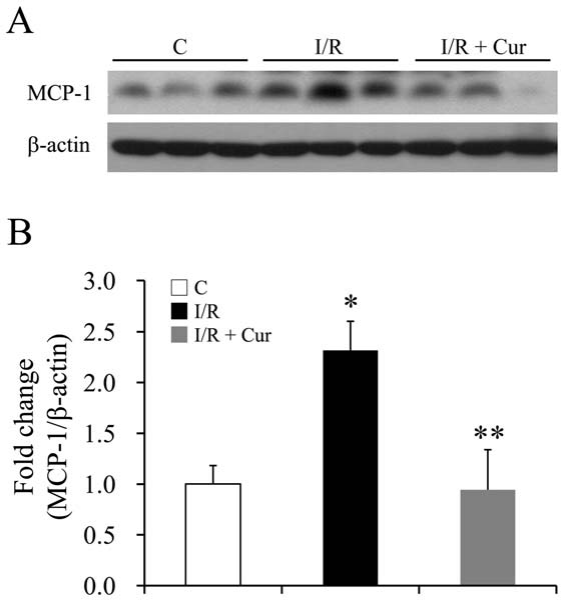
Curcumin inhibited retinal I/R induced up-regulation of MCP-1 12 hours after I/R injury. Representative Western blots of MCP-1 and β-actin expression levels in retinas (A) with densitometric quantitative results (B). Group abbreviations are: C, non-injured eyes; I/R, retinal I/R-injured eyes; I/R + Cur, I/R-injured eyes of animals treated with 0.05% curcumin 2 days before the injury. (n = 3–4 in each group; *P<0.05 compared with non-injured retinas; **P<0.05 compared with I/R-injured retinas). The error bars represent standard error of the mean.

The mRNA levels of several proinflammatory chemokines and cytokines also were investigated after retinal I/R injury. MCP-1 is a chemokine involved in the inflammatory response via recruitment of monocytes, memory T cells and dendritic cells to sites of tissue injury and infection [23]. We found that retinal I/R injury caused a 2.3-fold increase in the expression level of MCP-1, and oral administration of curcumin significantly inhibited this injury-induced over-expression of MCP-1 12 hours after the injury (Figure 9). The mRNA levels of pro-inflammatory cytokines, such as IL-1α, IL-1β, IL-6 and TNF-α, were significantly elevated 12 hours after the injury, but 0.05% curcumin pre-treatment did not inhibit the injury-induced up-regulation of these mRNA (data not shown).

## Discussion

In the present study, curcumin, a natural phenolic compound, was evaluated for its ability to inhibit neuronal and vascular degeneration induced by I/R injury in retinas. Our results indicate that pre-treatment with curcumin not only significantly inhibited neurodegeneration, but also inhibited retinal capillary degeneration (Figures 1 and 2). Interestingly, curcumin had a stronger protective effect on the vasculature than on retinal ganglion cells, and showed vascular protective effects even when administered after neurodegeneration was ongoing (Figure 1), suggesting that the vasoprotective effects of curcumin are independent of its neuronal protective effect.

Although the elimination half-life is short and the bioavailability is poor [24], curcumin has a variety of pharmacologic effects including acts as a neuroprotective agent in several neurologic diseases [11]. For example, curcumin inhibited neuronal cell death from dopaminergic neurotoxicity, apparently through the inhibition of c-Jun N-terminal kinase pathway [25], and it also protected the brain from I/R-induced neuronal damage by increasing the antioxidant system and decreasing oxidative stress [26], [27]. Furthermore, electroretinography studies demonstrated that curcumin significantly inhibited light-induced reductions in scotopic and photopic b-wave amplitudes in light-induced retinal degeneration, perhaps by inhibiting the activation of NF-κB and the over-expression of inflammatory genes [14].

A large range of curcumin doses and routes of administration have been used to treat neurodegenerative diseases in animal models (reviewed in [11]). Curcumin in diets (160–5000 ppm) has been shown to reduce amyloid and oxidative damage, and to inhibit the pathology in a transgenic mouse model of Alzheimer's disease [28], [29]. Dosages of curcumin comparable to those used previously by others significantly inhibited I/R-induced neurodegeneration including cell loss in GCL in the present study. Even though the brn3a staining and β-tubulin III western blot results demonstrated that curcumin has protective effects on ganglion cells, there are still possibilities that curcumin could protect also other neuronal cells in retina. Further experiments are still required to investigate the effects of curcumin on other cell types, such as displaced amacrine cell that consists about 50% neurons in the GCL [15].

NF-κB initiates transcription of many inflammatory genes, and activation of this transcription factor can occur by either canonical or non-canonical pathways. The canonical pathway of NF-κB activation involves the phosphorylation of IκBα, an endogenous inhibitory protein of NF-κB, by the kinase, IKKβ. This phosphorylation leads to IκBα degradation, translocation of NF-κB/p65∶p50 dimer to the nucleus, and subsequent activation of gene transcription [30]. In the non-canonical pathway, the NF-κB/RelB:p52 dimer is translocated into the nucleus through activation of IKKα [31]. Our results suggest curcumin might regulate both canonical and non-canonical ways of NF-κB activation, since administration of the drug to retinal I/R injured rats caused a modest up-regulation of IκBα and a dramatic down-regulation of p-IκBα (both consistent with its inhibitory effect on the canonical pathway), and inhibited IKKα expression (consistent with inhibition of the non-canonical pathway).

STAT3 is another inflammatory transcription factor responsive to cytokine signaling, such as the proinflammatory cytokine IL-6 [32]. Transcriptional activation of STAT3 involves its phosphorylation at Tyr705, which leads to the cascade of STAT3 dimerization, nuclear translocation and gene transcription [32]. On the other hand, phosphorylation of STAT3 at Ser727 regulates energy metabolism in cells [33]. STAT3 phosphorylation at the different sites is regulated by different kinases. In the present study, the observation that curcumin showed no effect on the phosphorylation levels of STAT3 at Ser727 (Figure 7) is consistent with our finding that the dosage of curcumin used by us had no effect on the activation of ERK (Figure 8). Our observation of curcumin-mediated inhibition of STAT3 phosphorylation at Tyr705 (Figure 7) is consistent with the previous report that curcumin inhibits constitutive and IL-6 inducible STAT3 activation [34]. Our results suggest that curcumin inhibits the STAT3 transcriptional activity via inhibition of Tyr705 phosphorylation, while having no effect on other cellular functions (such as energy regulation) that are mainly controlled by Ser727 phosphorylation [33]. Apparently, not all kinases that responsible for STAT3 phosphorylation can be regulated by curcumin.

Crosstalk between NF-κB and STAT3 has been found under many conditions [35]. Both NF-κB and STAT3 are rapidly activated in response to various stimuli including stresses and cytokines, although they are regulated by entirely different mechanisms. Once activated, NF-κB and STAT3 control the transcription of multiple inflammatory, proliferative and immune response genes, some of which even require the cooperation of these two transcription factors [36], [37].

MCP-1 is an inflammatory molecule [23] whose synthesis is regulated by NF-κB and STAT3 signaling pathways [36]. The up-regulation of MCP-1, which is responsible for the recruitment of inflammatory cells infiltration in the retina, has been reported after retinal I/R injury [3],[38]. In the present study, curcumin significantly inhibited the I/R-induced up-regulation of MCP-1 (Figure 9), consistent with its inhibitory effects on NF-κB and STAT3 activation (Figures 6 and 7).

Glial cells are important retinal cell types that modulate neuronal activity and affect the survival of neurons after various insults [39], [40]. Over-expression of GFAP is a recognized marker of glial activation, but the exact function of GFAP activation in retina remains unclear. Curcumin has been reported to down-regulate the elevated mRNA levels of GFAP after spinal cord injury [41], but showed no effect on retinal I/R-induced glial activation at the dose used in the present study. One possible explanation for this is that I/R induced GFAP overexpression in retina might be activated by pathways different from those in spinal cord injury.

In this study, we demonstrate that curcumin treatment results in a partial, but significant, inhibition of neuronal and vascular damage after I/R injury. Curcumin also inhibits multiple aspects of inflammation that are activated by the I/R injury, raising a possibility that inflammatory pathways might contribute to the inhibition of retinal neurovascular damage by the drug. Since neuronal and vascular damage are major elements in the pathogenesis also of several other retinal diseases, curcumin may thus serve as a promising candidate to inhibit such retinal pathology.
